# Endoplasmic Reticulum Stress Contributed to Dipyridamole-Induced Impaired Autophagic Flux and Glioma Apoptosis

**DOI:** 10.3390/ijms23020579

**Published:** 2022-01-06

**Authors:** Cheng-Yi Chang, Chih-Cheng Wu, Jiaan-Der Wang, Su-Lan Liao, Wen-Ying Chen, Yu-Hsiang Kuan, Wen-Yi Wang, Chun-Jung Chen

**Affiliations:** 1 Department of Surgery, Feng Yuan Hospital, Taichung 420, Taiwan; c.y.chang.ns@gmail.com; 2 Department of Veterinary Medicine, National Chung Hsing University, Taichung 402, Taiwan; wychen@dragon.nchu.edu.tw; 3 Department of Anesthesiology, Taichung Veterans General Hospital, Taichung 407, Taiwan; chihcheng.wu@gmail.com; 4 Department of Financial Engineering, Providence University, Taichung 433, Taiwan; 5 Department of Data Science and Big Data Analytics, Providence University, Taichung 433, Taiwan; 6 Children’s Medical Center, Taichung Veterans General Hospital, Taichung 407, Taiwan; wangjiaander@gmail.com; 7 Department of Industrial Engineering and Enterprise Information, Tunghai University, Taichung 407, Taiwan; 8 Department of Medical Research, Taichung Veterans General Hospital, Taichung 407, Taiwan; slliao@vghtc.gov.tw; 9 Department of Pharmacology, School of Medicine, Chung Shan Medical University, Taichung 402, Taiwan; kuanyh@csmu.edu.tw; 10 Department of Nursing, Hung Kuang University, Taichung 433, Taiwan; walice@sunrise.hk.edu.tw; 11 Department of Medical Laboratory Science and Biotechnology, China Medical University, Taichung 404, Taiwan

**Keywords:** apoptosis, autophagy, ER stress, glioma

## Abstract

Elevation of intracellular cAMP levels has been implicated in glioma cell proliferation inhibition, differentiation, and apoptosis. Inhibition of phosphodiesterase is a way to elevate intracellular cAMP levels. The present study aimed to investigate the anti-glioma potential of dipyridamole, an inhibitor of phosphodiesterase. Upon treatment with dipyridamole, human U87 glioma cells decreased cell viability, clonogenic colonization, migration, and invasion, along with Noxa upregulation, Endoplasmic Reticulum (ER) stress, impaired autophagic flux, Yes-associated Protein 1 (YAP1) phosphorylation, and YAP1 reduction. Pharmacological and genetic studies revealed the ability of dipyridamole to initiate Noxa-guided apoptosis through ER stress. Additionally, the current study further identified the biochemical role of YAP1 in communicating with ER stress and autophagy under situations of dipyridamole treatment. YAP1 promoted autophagy and protected glioma cells from dipyridamole-induced apoptotic cell death. Dipyridamole impaired autophagic flux and rendered glioma cells more vulnerable to apoptotic cell death through ER stress-inhibitable YAP1/autophagy axis. The overall cellular changes caused by dipyridamole appeared to ensure a successful completion of apoptosis. Dipyridamole also duplicated the biochemical changes and apoptosis in glioma T98G cells. Since dipyridamole has additional biochemical and pharmacological properties, further research centered on the anti-glioma mechanisms of dipyridamole is still needed.

## 1. Introduction

Glioma is the most common type of primary brain tumor, with the worst and most aggressive type of glioma being glioblastoma multiforme. Malignant glioma, particularly glioblastoma multiforme, shows highly proliferative, angiogenic, infiltrative, and invasive phenotypes. Their multiforme phenotypes are treatment obstacles for successful surgical resection and also causes of high recurrence rates [1,2]. Despite multimodal treatments and novel therapy being progressively developed, the clinical benefits of glioma patient treatment still remain unsatisfactory. The median survival period for patients with malignant glioma is approximately 1–2 years, with a 5-year survival rate of around 5–13% [3]. Therefore, advances in the elucidation of carcinogenic mechanisms, as well as developments of new compounds and novel therapeutic approaches targeting malignant glioma, are in high demand.

Malignant glioma carcinogenesis and its progression are complicated by panels of genetic amplification, mutation, and translocation, with results involving the activation of oncogenes and inactivation of tumor suppressor genes. Other than transmembrane growth factor receptors, defects in intracellular signaling pathways, including adenylyl cyclase, Ca^2+^, Phosphatidylinositol-3 kinase/phosphatase and Tensin/protein Kinase B/Mammalian Target of Rapamycin (PI3K/PTEN/Akt/mTOR), Signal Transducer and Activator of Transcription 3 (Stat3), Ras, p53, and Rb have all been identified in patients with malignant glioma [4,5,6]. The highlighted signaling pathways are theoretically surrogates being under consideration for therapeutic intervention against malignant glioma.

Adenylyl cyclase converts ATP into cyclic Adenosine Monophosphate (cAMP), a prototypical intracellular second messenger. The cAMP signaling pathway plays a fundamental role in cell proliferation, morphogenesis, migration, differentiation, and apoptosis. Deregulation of the cAMP signaling pathway has been revealed in malignancy, and has both promoting and inhibiting effects, depending upon cell types and environments [7,8]. In clinical specimens, the tissue contents of cAMP in brain tumors are lower than those in normal healthy tissues [9]. In vitro, elevation of intracellular cAMP levels inhibits glioma cell proliferation, triggers differentiation, induces apoptosis, and contributes to glioma’s cell-killing effects caused by anti-cancer treatment [5,10,11,12]. Since cAMP is synthesized by adenylyl cyclase and degraded by phosphodiesterase, the findings underscore the anti-glioma potential of adenylyl cyclase activators, cAMP analogues, and phosphodiesterase inhibitors.

Besides apoptosis, autophagy also plays crucial roles in cancer progression and inhibition [13]. Autophagy pathway is fully completed through different steps, including initiation, nucleation, elongation, maturation, fusion, and degradation. Among the key steps, the generation of a lipidated form of LC3, namely LC3-II, is a key component of autophagosome formation. SQSTM1/p62 is a cargo receptor recruiting cargo to autophagosome for degradation. The fusion of autophagosome and lysosome leads to autolysosome formation. Finally, the autophagic cargo, including LC3-II and p62, are degraded by lysosomal enzymes [13]. Since autophagy has cancer-promoting and cancer-suppressive effect, its diverse properties in cancer cells are emerging issues of cancer intervention.

Dipyridamole is one type of anti-platelet drug with pharmacological properties, including nucleoside uptake blockade and phosphodiesterase inhibition. Beyond those effects, dipyridamole also confers anti-cancer activity to fight against several types of malignancies, including glioma [14,15,16,17,18,19]. The disruption of Endoplasmic Reticulum (ER) homeostasis and the consequence of ER stress not only promotes glioma cell apoptosis but also sensitizes glioma cells to apoptotic treatment [20,21]. Although dipyridamole induces ER stress in hematological malignancies [16], the involvement and contribution of ER stress in dipyridamole-induced glioma apoptosis has not been reported. To extend the scope of anti-glioma studies regarding the cAMP signaling pathway, this study aimed to determine whether ER stress is a key component of apoptotic cell death in dipyridamole-treated glioma cells, as well as identifying any downstream surrogates which may contribute to the apoptotic program.

## 2. Results

### 2.1. Dipyridamole Showed Anti-Glioma Effects

**Figure 1:**
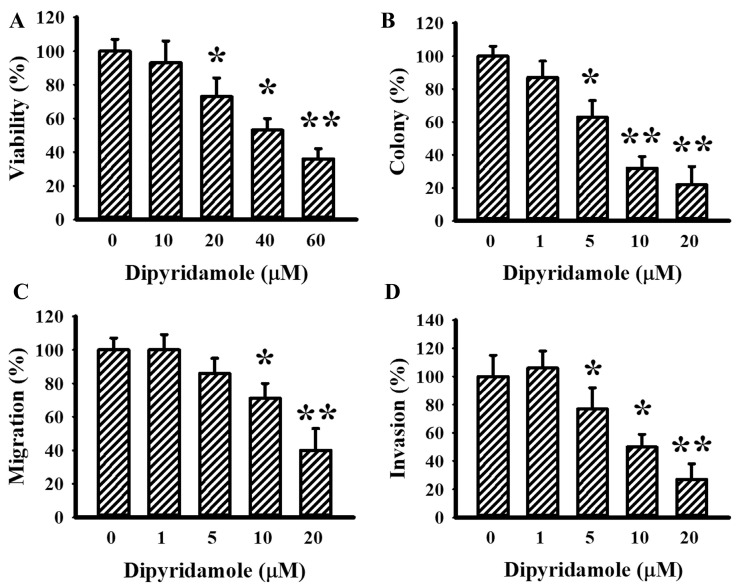
Dipyridamole decreased cell viability in U87 cells. U87 cells were treated with various concentrations of dipyridamole, as indicated. (**A**) Cell viability (24 h) was determined using MTS reduction assay. (**B**) Colony numbers (6 days) were visualized and counted. Cell migration (24 h) (**C**) and invasion (24 h) (**D**) were measured using a Transwell apparatus in the absence or presence of matrigel. * *p* < 0.05 and ** *p* < 0.01 vs. untreated control, *n* = 4.

To investigate the anti-glioma effects of dipyridamole, evaluations were performed on U87 glioma cells. Dipyridamole caused a reduction in both cell viability (Figure 1A) and clonogenic colony formation (Figure 1B). Using Transwell apparatus, both cell migration (Figure 1C and Supplementary Figure S1) and invasion (Figure 1D and Supplementary Figure S1) decreased upon dipyridamole exposure. These findings indicate that high concentrations of dipyridamole cause glioma cell viability loss, while low concentrations of dipyridamole have inhibitory effects on long-term cell growth, migration, and invasion.

### 2.2. Dipyridamole Caused Glioma Cell Apoptosis

**Figure 2:**
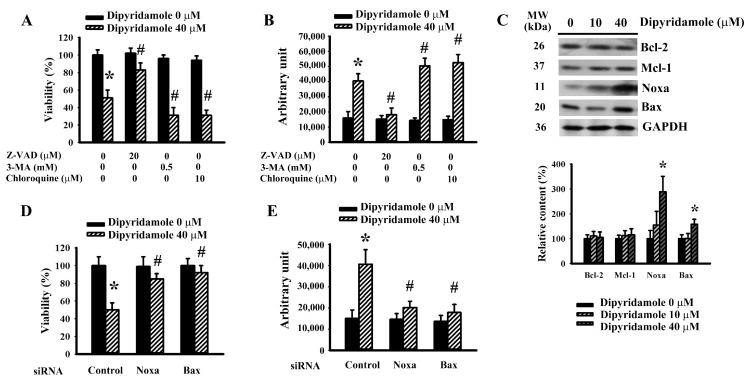
Dipyridamole elevated Noxa expression and caused apoptosis in U87 cells. U87 cells were treated with dipyridamole (0 and 40 μM) in the presence or absence of Z-VAD (20 μM), 3-MA (0.5 mM), and chloroquine (10 μM). (**A**) Cell viability (24 h) was determined using MTS reduction assay. (**B**) Caspase 3 activity (12 h) was measured using an enzymatic assay. (**C**) U87 cells were treated with various concentrations of dipyridamole, as indicated for 12 h. Proteins were determined using western blotting with indicated antibodies. Representative blots and quantitative results are shown. U87 cells were transfected with control siRNA, Noxa siRNA, and Bax siRNA for 24 h. The resultant transfected cells were treated with dipyridamole (0 and 40 μM). (**D**) Cell viability (24 h) was determined using an MTS reduction assay. (**E**) Caspase 3 activity (12 h) was measured using an enzymatic assay. * *p* < 0.05 vs. untreated control and # *p* < 0.05 vs. dipyridamole (40 μM) control, *n* = 4.

The identity of dipyridamole-induced glioma cell viability loss was explored through pharmacological and biochemical approaches. Toxicity curves of broad-spectrum caspase inhibitor Z-VAD-fmk (Supplementary Figure S2A) and autophagy inhibitors 3-Methyladenine (3-MA) (Supplementary Figure S2B) and chloroquine (Supplementary Figure S2C) were first determined in U87 cells. Sub-toxic concentrations of Z-VAD-fmk protected U87 cells against dipyridamole cytotoxicity, whereas 3-MA and chloroquine exacerbated cytotoxicity, as evidenced by the measurement of cell viability (Figure 2A) and caspase 3 activity (Figure 2B). Among the Bcl-2 family proteins, dipyridamole had promoting effects on the expression of Noxa and Bax, while displaying little effect on Bcl-2 and Mcl-1 (Figure 2C). To assess the potential involvement of Noxa and Bax in dipyridamole-induced apoptosis, RNA interfering was introduced. The cellular expression of both Noxa (Supplementary Figure S3A) and Bax (Supplementary Figure S3B) decreased when treated with each corresponding siRNA. Those Noxa-depleted and Bax-depleted cells were resistant to dipyridamole-induced viability loss (Figure 2D) and caspase 3 activation (Figure 2E). Current findings suggest that Noxa/Bax play an active role and offer an inhibitory property of autophagy in dipyridamole-induced glioma cell apoptosis.

### 2.3. Dipyridamole Induced Noxa Expression Involving ER Stress

**Figure 3:**
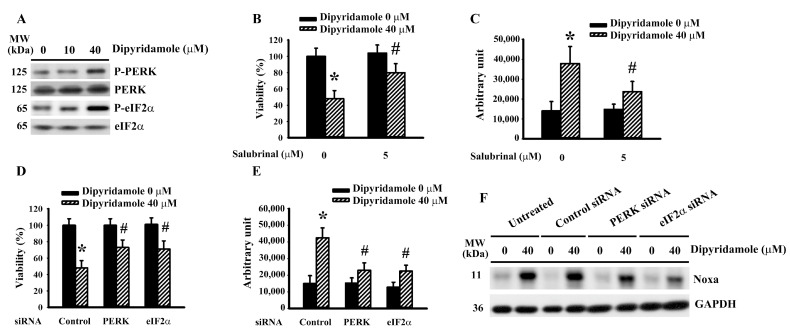
Dipyridamole induced ER stress in U87 cells. (**A**) U87 cells were treated with various concentrations of dipyridamole, as indicated for 12 h. Proteins were determined using western blotting with indicated antibodies. Representative blots are shown. U87 cells were treated with dipyridamole (0 and 40 μM) in the presence or absence of salubrinal (5 μM). (**B**) Cell viability (24 h) was determined using an MTS reduction assay. (**C**) Caspase 3 activity (12 h) was measured using an enzymatic assay. U87 cells were transfected with control siRNA, PERK siRNA, and eIF2α siRNA for 24 h. The resultant transfected cells were treated with dipyridamole (0 and 40 μM). (**D**) Cell viability (24 h) was determined using MTS reduction assay. (**E**) Caspase 3 activity (12 h) was measured using an enzymatic assay. (**F**) Proteins (12 h) were determined using western blotting with indicated antibodies. Representative blots are shown. * *p* < 0.05 vs. untreated control and # *p* < 0.05 vs. dipyridamole (40 μM) control, *n* = 4.

ER stress is a key component of glioma apoptosis and plays a regulatory role in Noxa expression [20,21]. Dipyridamole caused an elevation in phosphorylation in Protein Kinase R-like Endoplasmic Reticulum Kinase (PERK) and Eukaryotic Initiation Factor-2α (eIF2α) (Figure 3A and Supplementary Figure S4A), implying an occurrence of ER stress. An inhibitor of eIF2α and ER stress, salubrinal, alleviated dipyridamole-induced viability loss (Figure 3B) and caspase 3 activation (Figure 3C). Genetic silence of PERK (Supplementary Figure S4B) and eIF2α (Supplementary Figure S4C) rendered U87 cells less susceptible to dipyridamole-induced viability loss (Figure 3D) and caspase 3 activation (Figure 3E). Upon dipyridamole treatment, PERK-depleted and eIF2α-depleted cells decreased Noxa expression (Figure 3F and Supplementary Figure S4D). Therefore, a PERK/eIF2α-guided ER stress is pivotal to Noxa expression and apoptosis in dipyridamole-treated glioma cells.

### 2.4. Dipyridamole Impaired Autophagic Flux

**Figure 4:**
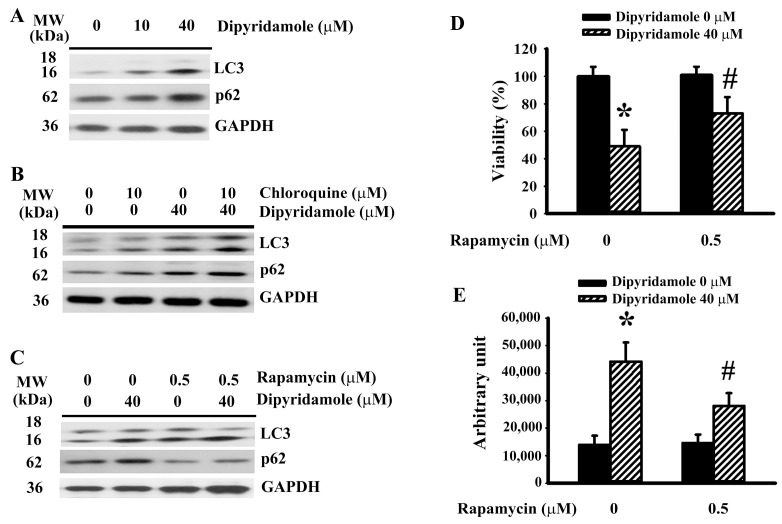
Dipyridamole impaired autophagic flux in U87 cells. (**A**) U87 cells were treated with various concentrations of dipyridamole, as indicated for 12 h. Proteins were determined using western blotting with indicated antibodies. Representative blots are shown. (**B**) U87 cells were treated with dipyridamole (0 and 40 μM) in the presence or absence of chloroquine (10 μM) for 12 h. Proteins were determined using western blotting with indicated antibodies. Representative blots are shown. U87 cells were treated with dipyridamole (0 and 40 μM) in the presence or absence of rapamycin (0.5 μM). (**C**) Proteins (12 h) were determined using western blotting with indicated antibodies. Representative blots are shown. (**D**) Cell viability (24 h) was determined using MTS reduction assay. (**E**) Caspase 3 activity (12 h) was measured using an enzymatic assay. * *p* < 0.05 vs. untreated control and # *p* < 0.05 vs. dipyridamole (40 μM) control, *n* = 4.

Beyond being a form of type II programmed cell death, autophagy has the ability to protect cells from apoptotic cell death [19,22,23,24]. p62 is a cargo adaptor of autophagy and substrate of autophagic flux [19]. Dipyridamole increased LC3-II generation and impaired p62 turnover (Figure 4A and Supplementary Figure S5A), implying an impairment in autophagic flux. To further demonstrate the impairment of autophagic degradation, the effects of lysosomal inhibition by chloroquine were investigated. Chloroquine caused an additional accumulation of LC3-II (Figure 4B and Supplementary Figure S5B) and p62 (Figure 4B and Supplementary Figure S5C) in dipyridamole-treated cells. On the contrary, autophagy inducer rapamycin [25] caused an additional generation of LC3-II (Figure 4C and Supplementary Figure S5D) and triggered p62 turnover (Figure 4C and Supplementary Figure S5E) in dipyridamole-treated cells. Moreover, rapamycin decreased dipyridamole-induced viability loss (Figure 4D) and caspase 3 activation (Figure 4E). Thus, dipyridamole impairs autophagic flux in glioma cells.

### 2.5. Dipyridamole Impaired Autophagic Flux Involving ER Stress-Inactivated Yes-Associated Protein 1 (YAP1)

**Figure 5:**
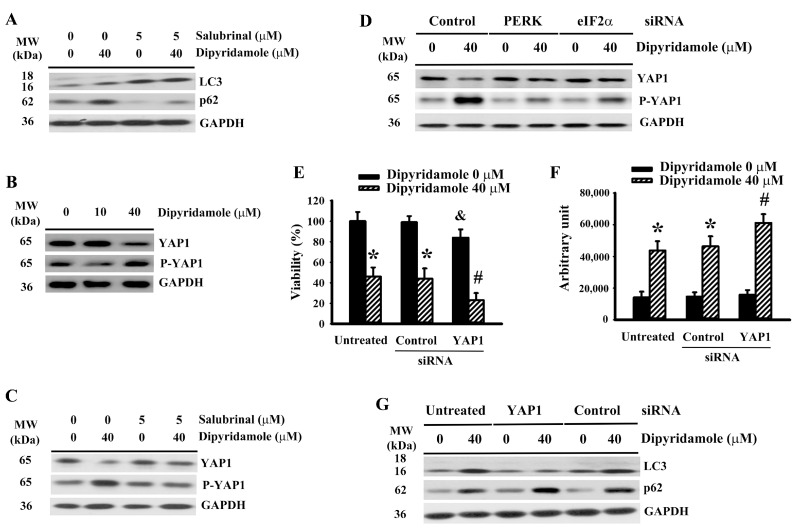
Dipyridamole increased YAP1 phosphorylation and decreased YAP1 content in U87 cells. (**A**,**C**) U87 cells were treated with dipyridamole (0 and 40 μM) in the presence or absence of salubrinal (5 μM) for 12 h. Proteins were determined using western blotting with indicated antibodies. Representative blots are shown. (**B**) U87 cells were treated with various concentrations of dipyridamole, as indicated for 12 h. Proteins were determined using western blotting with indicated antibodies. Representative blots are shown. (**D**) U87 cells were transfected with control siRNA, PERK siRNA, and eIF2α siRNA for 24 h. The resultant transfected cells were treated with dipyridamole (0 and 40 μM) for 12 h. Proteins were determined using western blotting with indicated antibodies. Representative blots are shown. U87 cells were transfected with control siRNA and YAP1 siRNA for 24 h. The resultant transfected cells were treated with dipyridamole (0 and 40 μM). (**E**) Cell viability (24 h) was determined using MTS reduction assay. (**F**) Caspase 3 activity (12 h) was measured using an enzymatic assay. (**G**) Proteins (12 h) were determined using western blotting with indicated antibodies. Representative blots are shown. * *p* < 0.05 vs. untreated control, # *p* < 0.05 vs. dipyridamole (40 μM) control, and ^&^ *p* < 0.05 vs. control siRNA/dipyridamole (0 μM), *n* = 4.

ER stress has diverse effects on apoptosis and autophagy [20,21,26,27]. Salubrinal had a positive effect on LC3-II generation (Figure 5A and Supplementary Figure S6A), and promoted p62 turnover (Figure 5A and Supplementary Figure S6B) in dipyridamole-treated U87 cells. Hippo component YAP1 is a promoter of autophagy, with the crosstalk between ER stress and YAP1-mediated autophagy being highlighted in many studies [28,29,30,31]. Dipyridamole increased YAP1 protein phosphorylation and decreased YAP1 protein levels (Figure 5B and Supplementary Figure S6C). The change in YAP1 protein levels (Figure 5C and Supplementary Figure S6D) and protein phosphorylation (Figure 5C and Supplementary Figure S6E) in dipyridamole-treated cells was reversed by salubrinal. Similar alterations were seen in PERK-depleted and eIF2α-depleted cells (Figure 5D, Supplementary Figure S6F,G). The genetic silence of YAP1 (Supplementary Figure S6H) exacerbated cell viability loss (Figure 5E) and caspase 3 activation (Figure 5F), as well as impairing LC3-II generation (Figure 5G and Supplementary Figure S6I) and p62 turnover (Figure 5G and Supplementary Figure S6J) in dipyridamole-treated cells. Our findings reveal that a reduction of YAP1 due to dipyridamole, involving the ER stress-mediated phosphorylatory mechanism, leads to impaired autophagic flux and augmented apoptosis in glioma cells.

### 2.6. Dipyridamole Displayed Universal Anti-Glioma Effects

To expand the anti-glioma effects of dipyridamole, T98G glioma cells were evaluated as well. Dipyridamole-treated T98G cells reduced cell viability (Supplementary Figure S7A), increased caspase 3 activity (Supplementary Figure S7B), and showed decreases in clonogenic colony formation (Supplementary Figure S7C), migration (Supplementary Figures S7D and S8), and invasion (Supplementary Figures S7E and S8). At the protein level, dipyridamole increased Noxa, PERK phosphorylation, eIF2α phosphorylation, LC3-II, p62, and YAP1 phosphorylation, while decreasing YAP1 (Supplementary Figure S7F). These results imply that dipyridamole shows universal anti-glioma effects.

## 3. Discussion

Dipyridamole is reported to have anti-cancer properties, including its effect on glioma. Proliferation inhibition and apoptosis are two common components of its anti-cancer capabilities [14,15,16,17,18,19]. Using *p53* wild type U87 cells and *p53* mutant T98G cells as study models, dipyridamole caused glioma cell apoptosis by involving the activation of the BH3-only protein Noxa. Sub-apoptotic concentrations of dipyridamole also conferred inhibitory effects on cell long-term growth, migration, and invasion. Intriguingly, dipyridamole impaired autophagic flux, resulting from YAP1 reduction. Autophagy activators protected glioma cells against dipyridamole apoptosis, while autophagy inhibitors exacerbated it. Both dipyridamole-induced Noxa activation and YAP1 reduction came from signaling control of the PERK/eIF2α ER stress. Therefore, ER stress is pivotal to dipyridamole-induced glioma cell death through the promotion of apoptosis and inhibition of apoptosis-antagonizing autophagy.

Apoptosis remains the main type of programmed cell death in cancer cell-killing. Although autophagy represents a form of type II programmed cell death, it also acts as a pro-survival strategy for escaping apoptosis. Autophagy causes glioma cell death and sensitizes glioma cells for treatments involving chemotherapy and radiotherapy [20,21,32]. However, protective autophagy is an alternative mechanism for the glioma cells to avoid apoptosis [24,33]. The complicated actions of autophagy on glioma cell fate depend upon both the types of treatment and cellular dynamics. In dipyridamole-treated glioma cells, autophagy highlighted a protective role since the promotion of autophagy by rapamycin alleviated apoptosis, while the inhibition of autophagy by 3-MA and chloroquine exacerbated apoptosis. The simultaneous impairment of autophagic flux appeared to be a concurrent strategy for dipyridamole in order to help ensure glioma apoptosis. Dipyridamole adopts the same strategy for avoiding the malignant progression of prostate cancer cells [19].

Cancer cells are proliferative and metabolically active, causing a heavy burden on ER homeostasis. Appropriate ER stress through the inducing of unfolded protein response, autophagy, and mitochondria crosstalk initiates panels of compensatory responses with an aim towards restoration of organelle’s physiological homeostasis, which is crucial to cancer cell malignant progression. Once unbalanced, uncontrolled, sustained, or chronic ER stress turns into a cell death signal through transcriptional or phosphorylatory mechanisms, it activates pro-apoptotic molecules and/or represses anti-apoptotic molecules. Noxa and CHOP are common transcriptional pro-apoptotic molecules resulting from uncontrolled ER stress, while the expression of anti-apoptotic Bcl-2 and Mcl-1 is downregulated upon sustained ER stress [20,21]. Unlike aspirin and indomethacin, which have an effect on Bcl-2, Mcl-1, and Noxa [20,21], Noxa appeared to be a susceptible Bcl-2 family protein upregulated by dipyridamole which contributed to glioma apoptosis. BH3-only Noxa protein selectively binds and inactivates Mcl-1. Glioma patients with a high expression of Mcl-1 are associated with tumor recurrence and a shorter survival period [34]. Experimental findings have indicated that Noxa activation is a crucial strategy for overriding Mcl-1-mediated apoptosis resistance [35,36]. Therefore, the imbalance between Noxa and Mcl-1 towards the former will guide the apoptotic program in dipyridamole-treated glioma cells.

YAP1 is an important transcriptional co-activator that is negatively regulated by the Hippo signaling pathway, playing a role in both organ size control and cell proliferation. Malignant glioma is associated with a high expression of YAP1. Clinical studies have revealed that YAP1 is a reliable prognostic biomarker and therapeutic target of glioma [37,38,39]. YAP1 also plays an active role in glioma cell proliferation, migration, and apoptosis under microRNA and long noncoding RNA control [40,41]. Increasing evidence has highlighted an additional biochemical role which YAP1 plays in autophagy, through either its transcriptional activation of autophagy components or its physical interaction for active autophagy complex assembly [42,43]. Additionally, YAP1 also plays a regulatory role in the maintenance of ER homeostasis. In the early adaptive phase of ER stress, unfolded protein response transcriptionally elevates YAP1 expression with an aim towards ER expansion and the alleviation of ER stress. Conversely, prolonged ER stress and the consequences of an overactivated PERK/eIF2α kinase axis will trigger YAP1 phosphorylation at Serine 127 residue, thus leading to degradation [28,31]. In dipyridamole-treated glioma cells, the levels of PERK/eIF2α phosphorylation paralleled with YAP1 phosphorylation and YAP1 reduction, while the pharmacological and genetic inhibition of ER stress alleviated dipyridamole-induced changes in YAP1. Moreover, the silencing of YAP1 caused further impairment in autophagic flux, as well as an exacerbation in caspase 3 activation and cell death upon dipyridamole treatment. A recent study has reported that imipramine impedes glioma progression with concurrent cAMP elevation and YAP1 inhibition [44]. We found that glioma cells with YAP1 depletion showed decreased viability without signs of caspase 3 activation. Therefore, YAP1 reduction-impaired autophagy is assumed to weaken protective adaption and ensure the apoptotic execution provoked by Noxa.

Although the current study provides interesting findings which expand on the dipyridamole anti-cancer effects centering on glioma apoptosis, there are limitations which should be addressed prior to any further implications. First, the primary targets and critical effectors have not yet been identified. In U87 glioma cells, the levels of cAMP increased from 1.2 pmol/mL to 13.4 pmol/mL upon treatment with dipyridamole (40 μM) (data not shown). However, the exact role elevated cAMP plays in dipyridamole-induced glioma apoptosis was not determined. Along with being an inhibitor of phosphodiesterase, dipyridamole also displays additional biochemical properties, including nucleoside uptake inhibition, free radical generation, and glutathione depletion [15,16,17]. Importantly, only cancerous glioma cells were included for investigation in this study. The cytotoxicity of dipyridamole towards normal non-cancerous cells will cause adverse effects. Thus, similar experiments should be duplicated in normal non-cancerous cells for comparison. To gain insight into its anti-glioma effects, those unsolved issues should be taken into consideration.

## 4. Materials and Methods

### 4.1. Cell Cultures

Human U87 MG glioblastoma (ATCC HTB-14) and T98G glioblastoma (ATCC CRL-1690) cells (American Type Culture Collection, Manasas, VA, USA) were maintained in Dulbecco’s Modified Eagle Medium (DMEM), with 10% Fetal Bovine Serum (FBS) supplementation according to our reported procedures [20,21,22]. All experiments were performed by placing cells in DMEM containing 2% FBS.

### 4.2. Cell Viability Assay

The cell viability of U87 and T98G cells was assessed using a MTS reduction-based assay kit in a 96-well plate (4 × 10^3^ cells/well) (CellTiter 96^®^ AQ_ueous_ Non-Radioactive Cell Proliferation Assay kit) according to the manufacturer’s instructions (Promega, Madison, WI, USA).

### 4.3. Clonogenic Colonization Assay

U87 and T98G cells were seeded onto 6-well plates at a density of 500 cells/well. Two days later, the cells were treated with various concentrations of dipyridamole (0–20 μM) for a period of 6 days. Cell colony was visualized through staining with crystal violet according to our reported procedures [21].

### 4.4. Cell Migration and Invasion Assay

Two hundred microliters of U87 and T98G cell suspension were seeded onto 24-well Transwell inserts at a density of 2 × 10^4^/200 μL. The lower chambers were filled with 600 μL of DMEM containing 10% FBS. The inserts were added with various concentrations of dipyridamole (0–20 μM) for a period of 24 h. The cells which transmigrated to the lower surface of the inserts were stained with crystal violet according to our reported procedures [45]. For the measurement of cell invasion, the inserts were pre-coated with Metrigel (24 μg/mL).

### 4.5. Caspase 3 Activity Assay

A Caspase Fluorometric Assay Kit (BioVision, Mountain View, CA, USA) was obtained for the measurement of caspase 3 activity according to the manufacturer’s instructions. The changes in fluorescence signals were measured with a fluorometer (E_x_ 380 nm and E_m_ 460 nm) and normalized with protein contents.

### 4.6. Small Interfering RNA (siRNA) Transfection

The delivery of siRNA (10 nM) into U87 cells was performed using Lipofectamine 2000 reagent (Invitrogen, Carlsbad, CA, USA) according to the manufacturer’s instructions and our reported procedures [21]. The siRNAs against human Noxa, Bax, PERK, eIF2α, and YAP1, as well as control siRNA were purchased from Santa Cruz Biotechnology (Santa Cruz, CA, USA).

### 4.7. Western Blot

The procedures for protein extraction, protein concentration determination, conventional SDS-PAGE, protein band visualization, and protein band intensity measurement were performed according to our reported methods [20,21]. The used antibodies which were recognized included Bcl-2 (1:1000), Mcl-1 (1:1000), Noxa (1:1000), Bax (1:1000), PERK (1:1000), eIF2α (1:1000), LC3 (1:1000), p62 (1:1000) (Santa Cruz Biotechnology, Santa Cruz, CA, USA), P-PERK (1:500, Threonine-982), P-YAP1 (1:500, Serine-127) (Abcam, Cambridge, UK), P-eIF2α (1:500, Serine-51), YAP1 (1:1000) (Cell Signaling, Danvers, MA, USA), and Glyceraldehyde-3-phosphate Dehydrogenase (GAPDH, 1:3000) (R&D Systems, Minneapolis, MN, USA).

### 4.8. Statistical Analysis

All data are presented as mean ± standard deviation. Two-way analysis of variance, followed by a Bonferroni or Tukey post-hoc test, was performed for group comparison using GraphPad Prism software version 5 (San Diego, CA, USA). A *p-*value less than 0.05 was considered statistically significant. A *p*-value less than 0.05 was marked with * and a *p*-value less than 0.01 was marked with **.

## 5. Conclusions

In conclusion, the present study provides additional insight into the anti-glioma mechanisms of dipyridamole, outlining its ability to suppress glioma cell viability, migration, and invasion. Moreover, our findings reveal the ability of dipyridamole to initiate Noxa-guided apoptosis through ER stress. Additionally, the current study also highlights the biochemical role which YAP1 plays in communicating with ER stress and autophagy under situations involving dipyridamole treatment. Dipyridamole impairs autophagic flux and renders glioma cells more vulnerable to apoptotic cell death through the ER stress-inhibitable YAP1/autophagy axis. The overall cellular changes caused by dipyridamole appear to ensure a successful completion of apoptosis. Despite these interesting findings, further insightful studies centered on the anti-glioma mechanisms of dipyridamole are still required.

## Data Availability

Not applicable.

## Supplementary Materials

The following supporting information can be downloaded at: https://www.mdpi.com/article/10.3390/ijms23020579/s1.
