# Supplementary material for: Endoplasmic Reticulum Stress Contributed to Dipyridamole-Induced Impaired Autophagic Flux and Glioma Apoptosis
Source: Int J Mol Sci. 2022 Jan 6;23(2):579. doi: 10.3390/ijms23020579 (PMC8775759; doi:10.3390/ijms23020579)
Supplement: Supplementary file 1 [file ijms-23-00579-s001.zip › ijms-1525059-supplementary.pdf]

International Journal of
Molecular Sciences

MDPI

Article

Endoplasmic Reticulum Stress Contributed to Dipyridamole-Induced Impaired Autophagic Flux and Glioma Apoptosis

Cheng-Yi Chang [1,2], Chih-Cheng Wu [3,4,5], Jiaan-Der Wang [6,7], Su-Lan Liao [8], Wen-Ying Chen [2], Yu-Hsiang Kuan [9], Wen-Yi Wang [10] and Chun-Jung Chen [8,11,*]

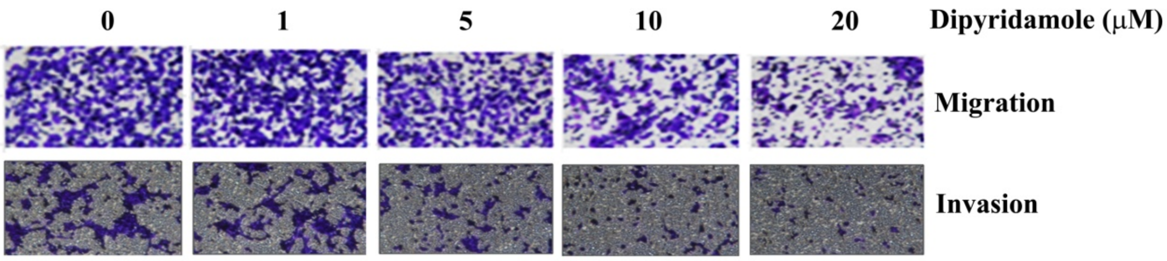

Figure S1. Dipyridamole decreased cell migration and invasion in U87 cells. U87 cells were treated with various concentrations of dipyridamole as indicated. Cell migration (24 hours) and invasion (24 hours) were measured using a Transwell apparatus in the absence or presence of matrigel. The cells which transmigrated to the lower surface of the inserts were stained with crystal violet. Representative images are shown. N = 4.

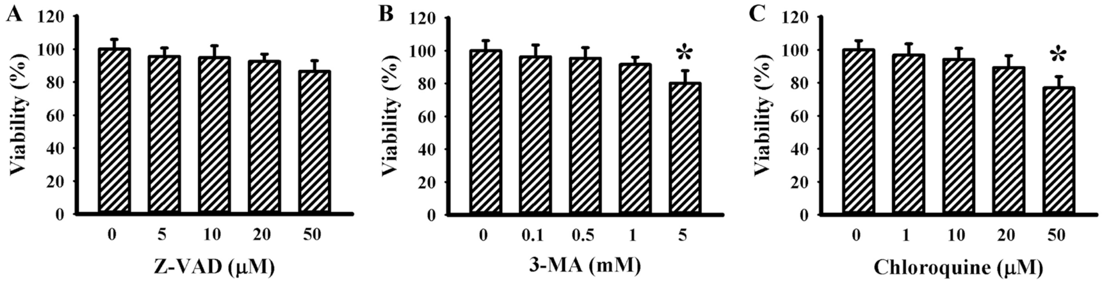

Figure S2. Toxicity curves were determined in U87 cells. U87 cells were treated with various concentrations of Z-VAD (**A**), 3-MA (**B**), or chloroquine (**C**) as indicated for 24 hours. Cell viability was determined using MTS reduction assay. *$p < 0.05$ vs. untreated control, $n = 4$.

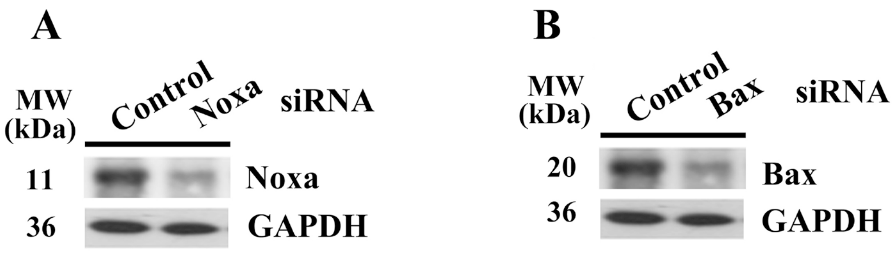

Figure S3. Genetic silencing of Noxa and Bax in U87 cells. U87 cells were transfected with control siRNA, Noxa siRNA (**A**), and Bax siRNA (**B**) for 24 hours. Proteins were determined using Western blotting with indicated antibodies. Representative blots are shown. N = 4.

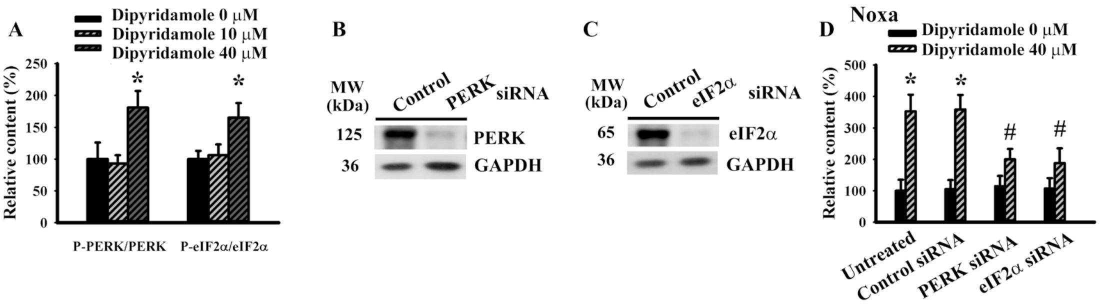

Figure S4. Dipyridamole induced ER stress in U87 cells. (**A**) U87 cells were treated with various concentrations of dipyridamole for 12 hours. Proteins were determined using Western blotting with indicated antibodies. Quantitative results are shown. U87 cells were transfected with control siRNA, PERK siRNA (**B**), and eIF2α siRNA (**C**) for 24 hours. Proteins were determined using Western blotting with indicated antibodies. Representative blots are shown. (**D**) The resultant transfected cells were treated with dipyridamole (0 and 40 μM). Proteins (12 hours) were determined using Western blotting with indicated antibodies. Quantitative results of Noxa are shown. **p* < 0.05 vs. untreated control and #*p* < 0.05 vs. dipyridamole (40 μM) control, *n* = 4.

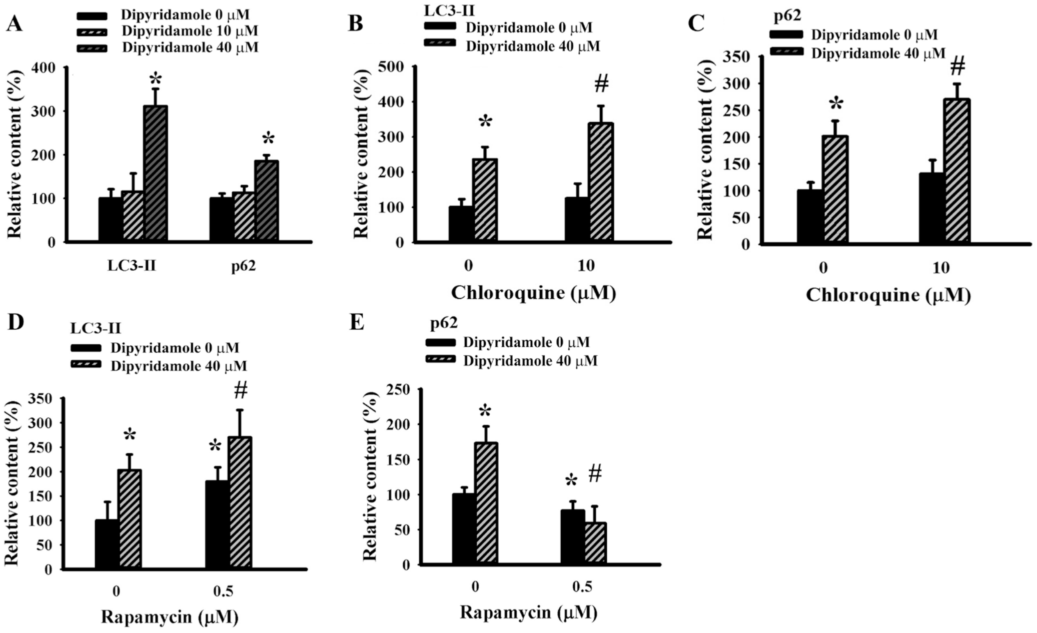

Figure S5. Dipyridamole impaired autophagic flux in U87 cells. (**A**) U87 cells were treated with various concentrations of dipyridamole as indicated for 12 hours. Proteins were determined using Western blotting with indicated antibodies. Quantitative results are shown. U87 cells were treated with dipyridamole (0 and 40 μM) in the presence or absence of chloroquine (10 μM). Proteins (12 hours) were determined using Western blotting with indicated antibodies. Quantitative results of LC3-II (**B**) and p62 (**C**) are shown. U87 cells were treated with dipyridamole (0 and 40 μM) in the presence or absence of rapamycin (0.5 μM). Proteins (12 hours) were determined using Western blotting with indicated antibodies. Quantitative results of LC3-II (**D**) and p62 (**E**) are shown. **p* < 0.05 vs. untreated control and #*p* < 0.05 vs. dipyridamole (40 μM) control, *n* = 4.

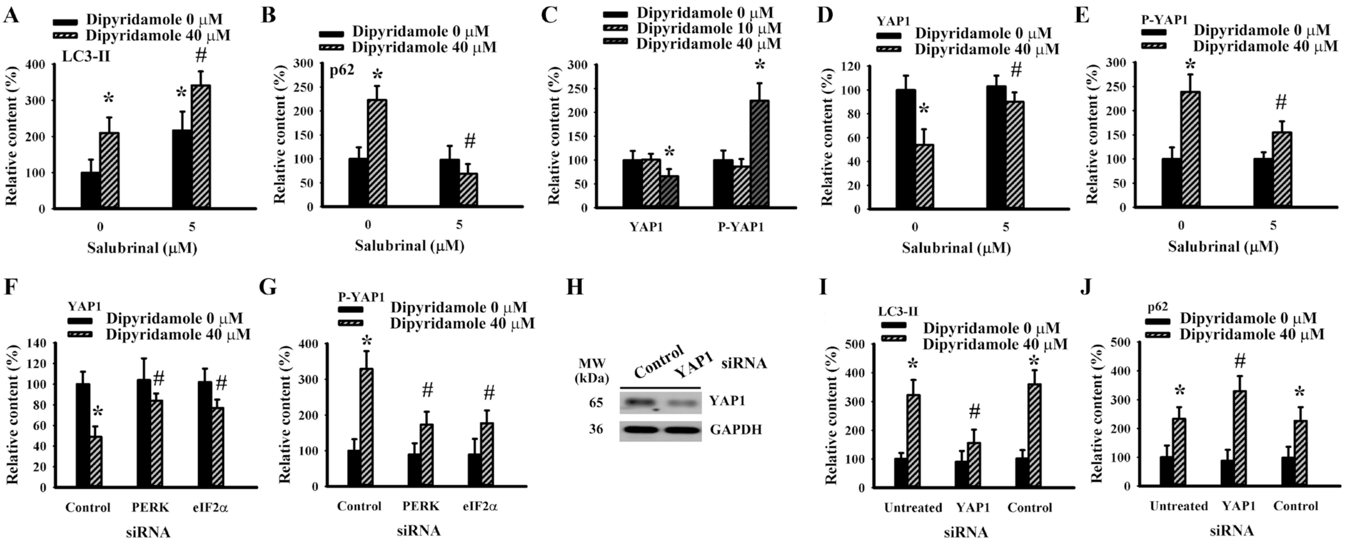

Figure S6. Dipyridamole increased YAP1 phosphorylation and decreased YAP1 content in U87 cells. U87 cells were treated with dipyridamole (0 and 40 μM) in the presence or absence of salubrinal (5 μM) for 12 hours. Proteins were determined using Western blotting with indicated antibodies. Quantitative results of LC3-II (**A**), p62 (**B**), YAP1 (**D**), and P-YAP1 (**E**) are shown. (**C**) U87 cells were treated with various concentrations of dipyridamole as indicated for 12 hours. Proteins were determined using Western blotting with indicated antibodies. Quantitative results are shown. U87 cells were transfected with control siRNA, PERK siRNA, and eIF2α siRNA for 24 hours. The resultant transfected cells were treated with dipyridamole (0 and 40 μM) for 12 hours. Proteins were determined using Western blotting with indicated antibodies. Quantitative results of YAP1 (**F**) and P-YAP1 (**G**) are shown. U87 cells were transfected with control siRNA and YAP1 siRNA for 24 hours. Proteins were determined using Western blotting with indicated antibodies. Representative blots are shown (**H**). The resultant transfected cells were treated with dipyridamole (0 and 40 μM). Proteins (12 hours) were determined using Western blotting with indicated antibodies. Quantitative results of LC3-II (**I**) and p62 (**J**) are shown. *$p < 0.05$ vs. untreated control, #$p < 0.05$ vs. dipyridamole (40 μM) control, $n = 4$.

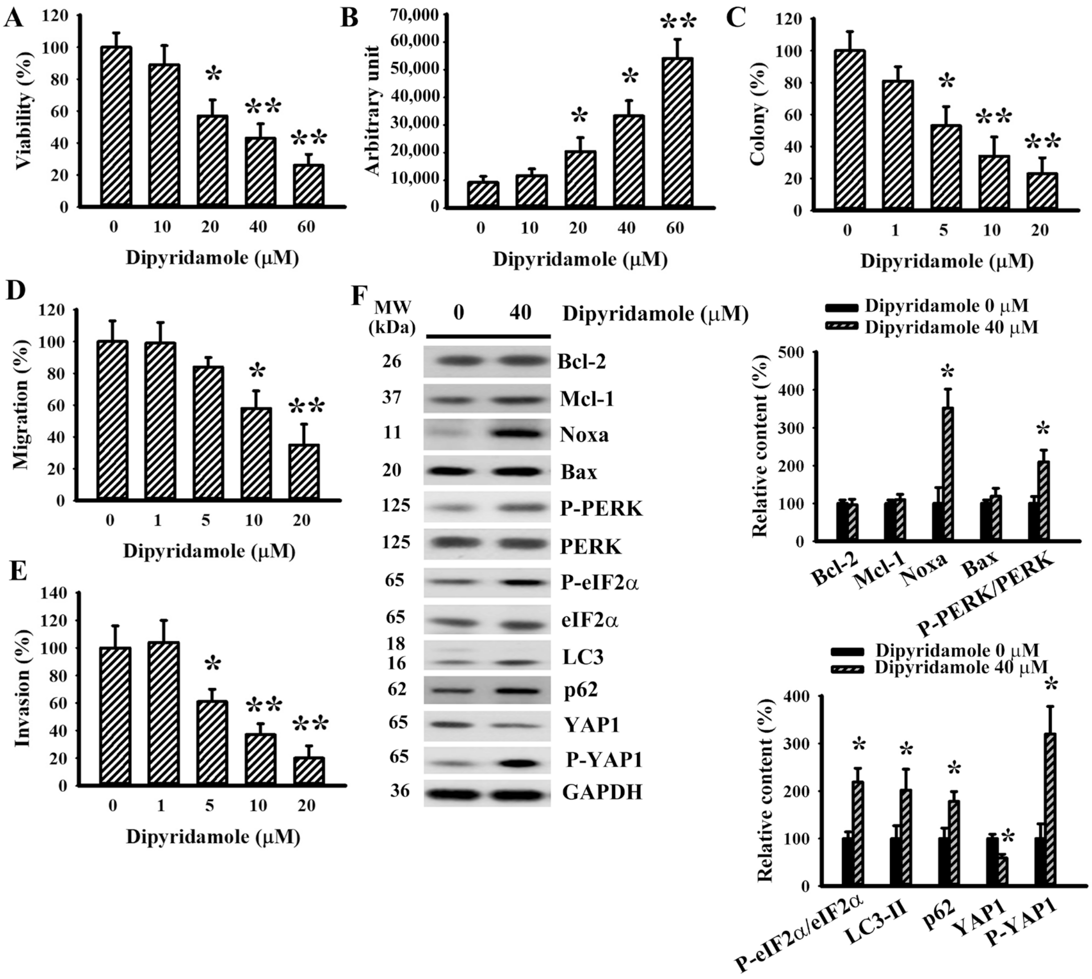

Figure S7. Dipyridamole caused apoptosis in T98G cells. T98G cells were treated with various concentrations of dipyridamole as indicated. (**A**) Cell viability (24 hours) was determined using MTS reduction assay. (**B**) Caspase 3 activity (12 hours) was measured using an enzymatic assay. (**C**) Colony numbers (6 days) were visualized and counted. Cell migration (24 hours) (**D**) and invasion (24 hours) (**E**) were measured using a Transwell apparatus in the absence or presence of matrigel. (**F**) T98G cells were treated with dipyridamole (0 and 40 μM) for 12 hours. Proteins were determined using Western blotting with indicated antibodies. Representative blots and quantitative results are shown. *$p < 0.05$ and **$p < 0.01$ vs. untreated control, $n = 4$.

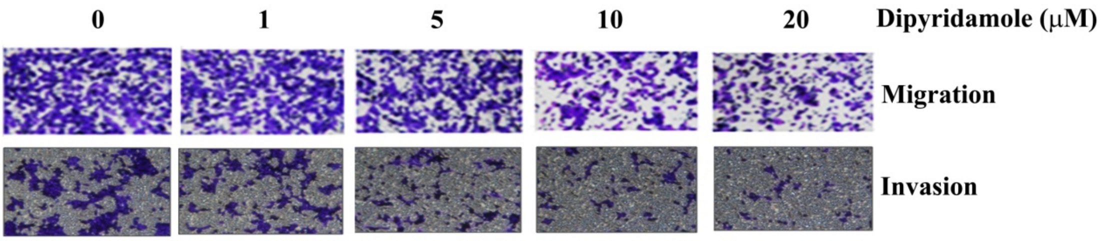

Figure S8. Dipyridamole decreased cell migration and invasion in T98G cells. T98G cells were treated with various concentrations of dipyridamole as indicated. Cell migration (24 hours) and invasion (24 hours) were measured using a Transwell apparatus in the absence or presence of matrigel. The cells which transmigrated to the lower surface of the inserts were stained with crystal violet. Representative images are shown.
